# Inorganic Arsenic in Rice-Based Beverages: Occurrence in Products Available on the Italian Market and Dietary Exposure Assessment

**DOI:** 10.3390/foods15020383

**Published:** 2026-01-21

**Authors:** Marilena D’Amato, Anna Chiara Turco, Teresa D’Amore, Francesco Vitale, Federico Marini, Paolo Stacchini, Angela Sorbo

**Affiliations:** 1 Department of Food Safety, Nutrition and Veterinary Public Health, Istituto Superiore di Sanità (Italian National Institute of Health), Viale Regina Elena 299, 00161 Rome, Italy; 2 Department of Chemistry, University of Rome la Sapienza, Piazzale Aldo Moro 5, 00185 Rome, Italy

**Keywords:** arsenic, HPLC-ICP-MS, speciation, inorganic arsenic, rice-based beverages, plant-based beverages, risk assessment, risk characterization, healthy diets, risk communication

## Abstract

Arsenic occurs in food in both inorganic (iAs) and organic (oAs) forms. Inorganic arsenic is highly toxic and classified as carcinogenic to humans, whereas oAs species, such as arsenobetaine (AB), monomethylarsonic acid (MMA), and dimethylarsinic acid (DMA), generally exhibit lower toxicity. Rice and rice-based products represent major contributors to dietary iAs exposure. Within this context, the present study provides an updated assessment of the occurrence of iAs and oAs in rice-based beverages available on the Italian market. A method for the simultaneous determination of iAs, AB, DMA, and MMA was developed and validated, and it exhibits adequate sensitivity to ensure robust occurrence data, eliminating left-censoring for iAs. A comprehensive analysis of twenty-five representative rice-based beverages was conducted, revealing that the contamination profiles exhibited a high degree of homogeneity, with iAs as the predominant species. All samples complied with the European maximum level for iAs in non-alcoholic rice-based beverages. When combined with recent Italian consumption data, these results enabled age-specific dietary exposure assessment. Although rice drinks contribute marginally to overall population exposure, estimated intakes for regular consumers in early childhood are associated with a small margin of exposure, raising potential concern for vulnerable subgroups. The increasing diversification of dietary habits and the rising consumption of plant-based beverages point to the necessity of continuous monitoring of iAs. Ongoing efforts in monitoring studies, updated food consumption surveys, and effective risk communication are essential to refine exposure assessment and thereby enhance public health protection.

## 1. Introduction

Arsenic is a metalloid ubiquitous in the environment as both inorganic and organic species, occurring naturally and also as a result of human activities. Inorganic arsenic (iAs) mainly comprises arsenite (As(III)) and arsenate (As(V)), representing the trivalent and pentavalent oxidation states, respectively. These two species are considered the most toxic forms of As and show comparable potency, although As(III) generally displays slightly higher toxicity due to its greater reactivity toward cellular thiols and its easier translocation across biological membranes [1,2].

On the other hand, organoarsenical compounds (oAs), which include arsenobetaine (AB), arsenocholine (AC), monomethylarsonic acid (MMA), dimethylarsinic acid (DMA), arsenosugars, and arsenolipids, are generally characterized by substantially lower toxicity [3]. AB and AC, together with several arsenosugars, are the predominant species in seafood and seaweeds, while MMA and DMA may be found in plant-based matrices as intermediate products of microbial or plant metabolism [4,5].

Thus, iAs species have been classified by the International Agency for Research on Cancer (IARC) as “carcinogenic to humans” (Group 1). More recently, the IARC classified DMA and MMA as “possibly carcinogenic to humans” (Group 2B) and AB and other oAs compounds, as “not classifiable as to their carcinogenicity to humans” (Group 3) [6].

Following the request of the European Commission (EC), based on newly available epidemiological studies, the European Food Safety Authority (EFSA) in 2024 updated the human health risk assessment of iAs in food. A Reference Point (RP) of 0.06 μg iAs/kg bw per day was identified by EFSA experts based on the benchmark dose lower confidence limit corresponding to the 5% increase of the background incidence for skin cancer after adjustment for confounders (BMDL05) [7].

Food and drinking water represent the main routes of exposure to iAs for the general European population. Rice and rice-based products are among the main contributors across these populations [8]. Rice (*Oryza sativa* L.), in fact, accumulates significantly higher levels of iAs than other cereals due to its nature and the anaerobic growth conditions in rice fields. It is a staple food in many South-Eastern Asian countries, but it is also increasingly consumed in Western countries due to its bland taste, nutritional value, absence of gluten, and relatively low allergic potential [9,10,11,12].

In 2021, considering the newly available occurrence data for iAs in food, EFSA revised the dietary exposure assessment to iAs and identified rice drinks as products deserving attention when assessing dietary exposure, due to the increasing consumption as a milk alternative in certain specific population group such as vegans/vegetarians, people suffering from coeliac disease or lactose intolerance, and individuals who avoid hormones in animal milks due to cancer-related illness. Moreover, limited consumption data and high uncertainty linked to left-censored measurements represented other limitations [13]. In light of that information, the EC has set a new maximum level of 0.030 mg kg^−1^ for iAs in non-alcoholic rice-based drinks and EFSA recommended the collection of new consumption data for specific populations and the use of validated analytical methods with adequate sensitivity [14].

In this framework, the aim of the present study was (i) to generate robust and up-to-date occurrence data on inorganic and organic arsenic species in rice-based beverages available on the Italian market (ii) to characterize contamination profiles and inter-species distribution, and (iii) to assess dietary exposure and risk for different age groups of the Italian population, with particular attention to vulnerable consumers.

The method for the determination of iAs and oAs species (AB, DMA and MMA) was developed and validated in accordance with UNI EN 16802:2016 [15]. In parallel, total arsenic (tAs) was quantified by inductively coupled plasma-mass spectrometry (ICP-MS). The validated method was applied to 25 rice-based beverages collected to be representative of the Italian market. The resulting occurrence dataset revealed a generally homogeneous contamination profile, and the findings were compared with data reported by EFSA and published in recent international studies on similar matrices. The present study was carried out within the framework of the activities of the Italian National Reference Laboratory for metals and nitrogenous compounds in food (NRL-MN).

Based on the analytical results and the most recent Italian consumption data, dietary exposure to iAs from rice drinks was estimated and the risk was characterized in the general population, consumers, and subgroups using the margin of exposure approach. The outcome indicates a potentially concerning scenario for certain population groups. Although the risk appears insufficiently controlled, the data clearly highlight the need for updated and robust consumption information, particularly in view of evolving dietary patterns and the growing use of plant-based beverages among specific subgroups of the population.

## 2. Materials and Methods

### 2.1. Chemicals and Reagents

Ultrapure grade water was used throughout this study (18.3 MΩ cm, Zeener UP 900 Scholar UV, Human Corporation, Seoul, Korea).

Suprapure nitric acid (HNO_3_) 67–69% *v*/*v* (ROMIL Ltd., Waterbeach, Cambridge, UK) was used for the mineralization procedure, while ultrapure HNO_3_ (67–69% *v*/*v* ROMIL Ltd., Waterbeach, Cambridge, UK) was used for the preparation of the extraction mixtures. Hydrogen peroxide (H_2_O_2_) used in both extraction and mineralization is of suprapure grade (30% *v*/*v*, ROMIL Ltd., Waterbeach, Cambridge, UK).

The calibration and internal standard solutions of rhodium and arsenic used for the determination of tAs were obtained from certified rhodium (Rh) standard (1000 ± 5 mg L^−1^, High Purity Standard, Charleston, SC, USA) and As (1000 mg L^−1^, CPAchem Ltd., Bogomilovo, Bulgaria).

Standard solutions of the 1 mg L^−1^ arsenical species (AB, MMA, DMA) used in the speciation analysis were obtained by dissolution in water of AB (Fluka-Merck KGaA, Darmstadt, Germany), MMA (Tri Chemical Laboratories Inc., Yamanashi, Japan), and DMA (≥99.0% Sigma-Aldrich-Merck KGaA, Darmstadt, Germany), respectively. The solution of As(V) was obtained from the As^+5^ standard (999 ± 5 µg mL^−1^, Inorganic Ventures, Christiansburg, VA, USA). Solutions of the arsenical species were stored in the dark at −20 °C to prevent possible interconversion between the different species, and their concentration was verified by ICP-MS analysis. Standard solutions were daily diluted with the extraction solution for appropriate matrix matching and subjected to speciation analysis by high-performance liquid chromatography coupled with inductively coupled plasma mass spectrometry (HPLC-ICP-MS) to verify their chromatographic purity.

Ammonium carbonate (NH_4_)_2_CO_3_ (99.999% Sigma-Aldrich, Merck KGaA, Darmstadt, Germany), ammonia solution (25% *v*/*v* Suprapur^®^, Merck KGaA, Darmstadt, Germany), and methanol CH_3_OH (HPLC grade, Panreac Quimica S.A., Barcelona, Spain) were used for the preparation of the mobile phase used for speciation analysis. Prior to use, the mobile phase was filtered with the Millipore^®^ Stericup^®^ vacuum filtration system equipped with a Millipore (Durapore^TM^) 0.22 μm membrane filter (VWR International, Milan, Italy) and degassed with a NEXAR Channel Vacuum Degasser (PerkinElmer, Shelton, CT, USA).

### 2.2. Reference Materials and Samples

Twenty-five samples of rice-based drinks were purchased between April 2022 and March 2023 from various retail outlets to ensure representativeness of products commonly available on the Italian market. All rice drink samples contain water, rice (at varying percentages), sunflower oil, and sea salt. Two products were fortified with vitamins (vit B2, vit B12, vit D), while guar gum and gellan gum were added as stabilizers and texturizing agents in 4 samples. No specific information on the origin of the rice was provided on product labels, except for rice of Italian origin (48% of samples). A post-sampling analysis revealed that the majority of products (88%) available on the Italian retails were certified organic. The rice-based drinks were stored at room temperature prior to opening and at −20 °C thereafter.

NIST rice flour SRMs 1568b (National Institute of Standards and Technology, Gaithersburg, MD, USA), NMIJ 7532-a (National Metrology Institute of Japan, Tsukuba, Japan) and ERM-BC211 (Joint Research Centre, Institute for Reference Materials and Measurements, Geel, Belgium) were used as certified reference materials (CRMs) for As species and tAs determination.

### 2.3. Instrumentation

All analytical determinations were performed by a NexION^®^ 2000 Inductively Coupled Plasma Mass Spectrometer (PerkinElmer, Shelton, CT, USA) equipped with a nickel Triple Cone Interface, a Meinhard quartz concentric nebulizer, and a quartz cyclonic spray chamber. For speciation analysis, a NexSAR™ 200 inert HPLC pump was used. Syngistix™ (version 3.3) for ICP-MS and Clarity (version 8.1) (PerkinElmer, Shelton, CT, USA) software programs were used for total and speciation analyses, respectively. Operational conditions are reported in Table 1.

### 2.4. Total Arsenic Determination

Total arsenic was determined by ICP-MS, according to the European Standard UNI EN17851:2023 [16], following closed vessel microwave-assisted digestion performed using a Milestone ultraWAVE microwave system (FKV, Bergamo, Italy) equipped with a Teflon 15-vessel rotor.

Approximately 1.0 g of each sample (0.3 g ca. for CRMs) was weighed into a Teflon vessel and added with 3 mL of HNO_3_, 3 mL of H_2_O, and 1 mL of H_2_O_2_. Samples were subjected to a microwave digestion program with a temperature increase to 200 °C over 40 min, followed by a holding period at this temperature for additional 25 min.

After cooling at room temperature, the digested samples were transferred into polypropylene tubes and diluted up to 20 mL with water. The tAs concentration was determined after appropriate dilution using the collision mode (KED) for the removal of ArCl interference. Quantification was performed by external calibration (1% HNO_3_) with rhodium (1 mg L^−1^) as internal standard.

### 2.5. Arsenic Speciation

The method used for iAs was based on the European Standard UNI EN 16802:2016 for the determination of iAs in foodstuffs of marine and plant origin by anion-exchange HPLC-ICP-MS [15] after a mild acid water bath extraction. This analytical method provides complete oxidation with quantitative conversion of As(III) to As(V), without conversion of other oAs into iAs, so that the quantification of As(V) corresponds to iAs (sum of As(III) and As(V)), which appears as a well-separated peak in the HPLC-ICP-MS chromatogram, with an improvement in the detection sensitivity for iAs. ArCl interference was resolved chromatographically. The standard mode for ICP-MS determination was used. Briefly, 1.0 g of each rice drink sample (0.3 g ca. for CRMs) was treated with a solution of 0.1 M HNO_3_ and 3% H_2_O_2_ in a heated water bath at 90 °C ± 2 °C under gentle agitation for 60 min ± 5 min.

After cooling, the extracts were centrifuged for 15 min at 8000 rpm and 4 °C, and the supernatants filtered with PVDF 0.22 μm filters.

### 2.6. Dietary Exposure Assessment

Dietary exposure to iAs from rice-based drinks was estimated by combining the occurrence data generated in this study with individual food consumption data from the most recent Italian national dietary surveys (IV SCAI CHILD and IV SCAI ADULT), as included in the EFSA Comprehensive European Food Consumption Database (update: December 2022) [17,18]. For the total dietary exposure to iAs in the Italian population, the results of the latest EFSA chronic dietary exposure assessment were used [8].

To ensure accurate matching between consumption records and analytical data, the FoodEx2 classification system was applied. Occurrence values for iAs were assigned to the corresponding FoodEx2 code along the following hierarchical pathway.

Exposure hierarchy: (L1): Products for non-standard diets, food imitates, and food supplements → (L2): Meat and dairy imitates → (L3): Dairy imitates → (L4): Milk imitates → (L5): Rice drink. Only consumption events explicitly coded as “Rice drink” at the L5 level were included [19].

A deterministic chronic exposure assessment was conducted following EFSA standard methodology. For each subject, daily intake of rice drinks (g day^−1^) was expressed on a body weight basis (g kg^−1^ bw per day), using the individual body weight recorded in the survey. Exposure was estimated by multiplying the consumption amount by the mean concentration of iAs measured in rice drink samples. Exposure calculations were performed for both (i) all subjects, including non-consumers (zero-consumption on recording days), and (ii) consumers only, defined as individuals reporting at least one consumption event of rice drink [13].

Given the absence of censored values in the analytical dataset, no substitution approach (lower bound (LB)/middle bound (MB)/upper bound UB) was required for the rice drink occurrence. For comparison with overall dietary exposure, values from EFSA 2021 chronic exposure assessment were used (Annex B EFSA 2021) [8], expressed as LB and UB estimates. Exposure estimates were generated for the population groups defined in the Comprehensive Database: toddlers (12–35 months), other children (3–9 years), adolescents (10–17 years), adults (18–64 years), and the elderly (≥65 years). Mean chronic exposure was used as the relevant metric for risk characterization, in line with EFSA guidance for substances with long-term effects.

### 2.7. Risk Characterization

Risk characterization was performed using the margin of exposure (MOE) approach, as recommended by EFSA for substances that are both genotoxic and carcinogenic, such as iAs. The MOE is the ratio between the RP (the dose at which a low but measurable adverse effect is observed) and the human intake, and therefore it makes no implicit assumptions about a “safe” intake [20]. In its most recent report on the risk assessment of iAs in food, EFSA attempted, for the first time, to identify an acceptable MOE that did not raise concerns. Notwithstanding the Panel decision to abstain from assigning a value to an MOE of low concern, it was determined that the probability of exceeding the BMD, estimated to be 5%, would result in an MOE lower than 1, thereby indicating a potential for concern. This indicative value was estimated by the means of both a deterministic approach, where each selected critical effect and exposure scenario was delineated, and a probabilistic approach, employing the Monte Carlo method [7].

## 3. Results and Discussion

### 3.1. Method Validation for Inorganic Arsenic Determination

**Figure 1:**
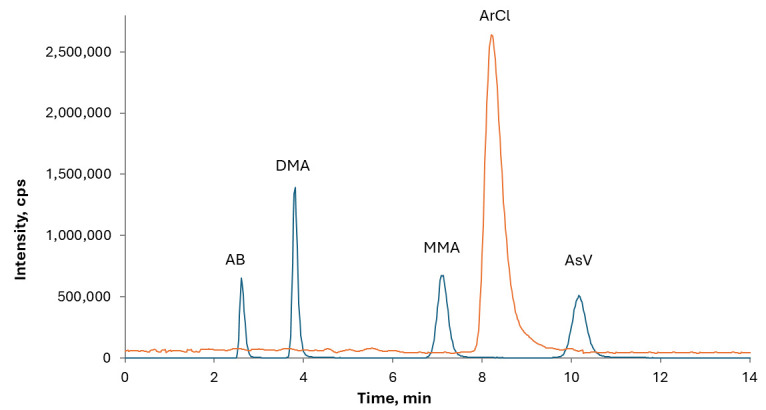
Typical chromatogram of a mixture of As species standards at a concentration of 10 μg kg^−1^. The orange trace corresponds to *m*/*z* 35 (Cl).

The optimal chromatographic conditions, as recommended by the Standard UNI EN 16802:2016, were established by verifying the sufficient separation of the arsenical species of interest, namely AB, MMA, DMA, and As(V). The absence of coelution of arsenate and chloride peaks was verified to avoid possible interference from the polyatomic ion ^40^Ar^35^Cl^+^ on ^75^As, and the standard mode determination was chosen for speciation analysis. As shown in Figure 1, the chloride peak is well separated from iAs, indicating that potential interference from ArCl can be disregarded and standard mode determination considered appropriate.

The limit of detection (LOD) and limit of quantification (LOQ) for iAs were calculated using the 3 σ and 10 σ criterion, respectively, where σ is the standard deviation of ten independent extraction blank analyses spiked at a level of 0.5 μg kg^−1^. The calculated LOQs of the method were 0.7 and 1.5 μg kg^−1^ for fresh and dried samples, respectively. Similarly, for fresh samples, the LOQs of the method for DMA and MMA were 0.5 and 0.4 μg kg^−1^, respectively.

Precision was assessed in terms of repeatability and intermediate precision (within-laboratory reproducibility) by determining within-day and between-day (2 days) relative standard deviations (RSDs) [21,22] from six independent replicates at three levels of concentration. In particular, two rice-based CRMs, namely ERM-BC211 and NMIJ CRM 7532-a, as well as a sample of the rice drink spiked at level of interest, i.e., 0.030 mg kg^−1^, were used. The repeatability and intermediate precision, expressed as RSD ranged from 0.7 to 4.7% and from 2.2 to 8.1%, respectively. The validation parameters for iAs comply with the requirements laid down in Regulation (EC) No 333/2007 [23]. In particular, the LOD and LOQ values for rice-based beverages are well below the maximum values permitted by the Regulation (i.e., 9 μg kg^−1^ and 30 μg kg^−1^ for LOD and LOQ, respectively). Furthermore, for both repeatability and intermediate precision, the HorRat ratio—defined as the ratio between the observed RSD and the RSD calculated using the Horwitz equation—is well below 2, confirming values lower than the limit required by the Regulation. In accordance with the International Standard ISO 21748:2017, expanded uncertainty was estimated by considering the reproducibility derived from interlaboratory validation studies as the main contributing factor; in this case the expanded uncertainty is calculated as two times the relative standard deviation reproducibility (RSDR) of the standard method UNI EN 16802:2016, considering a matrix similar to that of interest [15,24].

**Figure 2:**
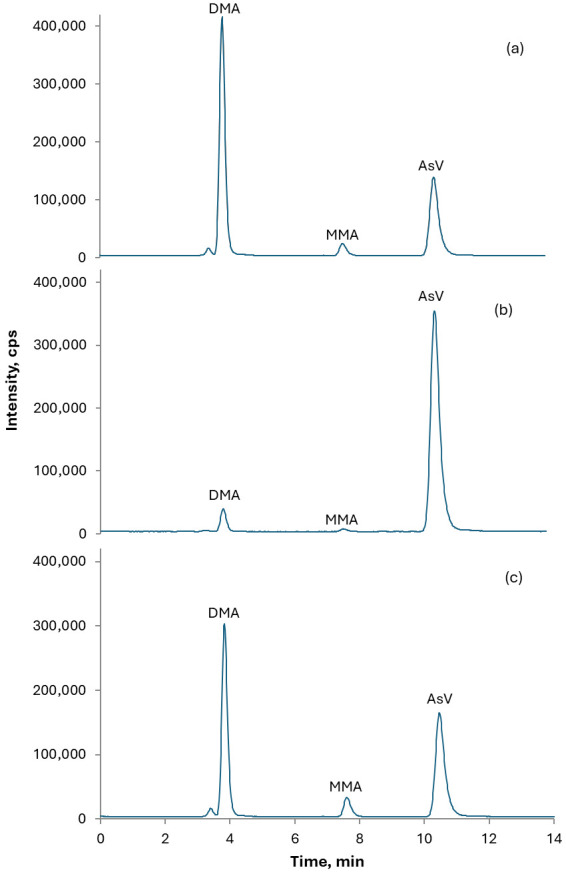
Chromatograms of CRMs extracts used in the study: (**a**) NIST SRM 1568b; (**b**) NMIJ CRM 7532-a; (**c**) ERM-BC211.

Rice CRMs (NIST SRM 1568b, ERM-BC211, and NMIJ CRM 7532-a) were used throughout the study for trueness assessment. The chromatographic profiles obtained for the CRMs are presented in Figure 2. The results show good agreement with the certified values for both tAs and iAs. Results for MMA and DMA are also reported, revealing good accordance with certified values, despite the relatively low certified concentration values for MMA (Table 2). Mass balance, as ratio of the sum of As species in the extract to tAs, was also reported as a measure of both extraction efficiency and column recovery. Spiking/recovery experiments were also performed using a rice drink sample spiked with 20 μg kg^−1^ iAs to evaluate trueness for iAs in the matrix at the level of interest [21,22].

### 3.2. Occurence of Inorganic Arsenic and Organo-Arsenic Species in Rice-Based Beverages

The present study investigates the contamination pattern of iAs and oAs species in rice-based beverages. The sampling strategy reflects the products currently available on the Italian market and was designed to provide a representative picture of the situation across the entire country. Overall, twenty-five samples were analyzed, including sixteen samples containing rice as sole ingredient, two brown rice drinks, four rice-and-coconut drinks, two rice-and-almond drinks, and one beverage containing rice, hazelnut, and cocoa. Table 3 reports the concentration values of tAs, iAs and oAs, together with the rice content (%) and the percentage of iAs compared with the sum of As species. In addition, mean, median, and range (min-max) concentrations are reported.

**Figure 3:**
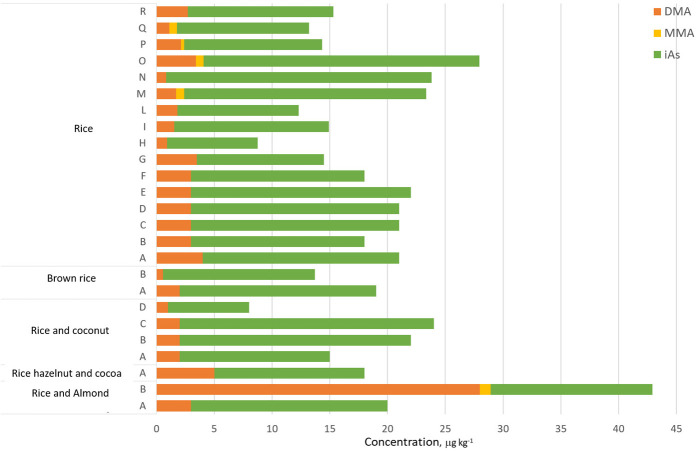
Distribution of As species in rice drinks available on the Italian market.

The concentrations observed were homogenous, as well as the distribution profiles of As species (Figure 3), and iAs was the most representative species, with quantifiable levels in 100% of the samples.

The average percentage of iAs relative to the sum of the species was 84%, ranging from 33% to 97%, in line with the literature data [8]. The mean iAs and DMA concentrations were found to be 15 µg kg^−1^ and 3 µg kg^−1^, respectively. MMA was found at low levels in six samples, consistent with its molecular instability (it is gradually converted to DMA, which is more stable) across vegetable food matrices, as well as with its limited translocation into rice from soil [1,25]. Notably, one sample of rice and almond drink exhibited a higher content of oAs (28 and 9 µg kg^−1^ of DMA and MMA, respectively). Despite the findings of other recent studies which concluded that almond-based imitation milks may be considered among the safest options in terms of iAs content, the limited available data and the presence of other ingredients suggest further consideration is required [26,27].

In the analyzed samples, the recovery ranged from 74% to 113%, which was satisfactory compared to the acceptance criteria set out by the Commission Decision 2002/657/EC concerning the performance of analytical methods [28].

**Figure 4:**
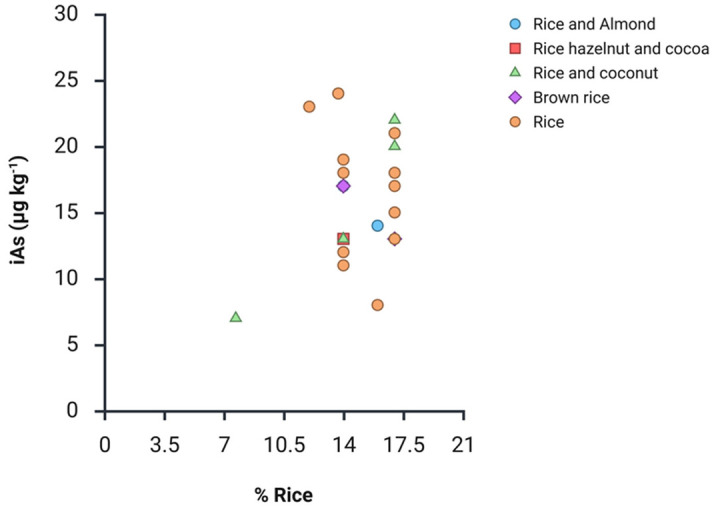
Scatter plot of declared rice content (%) and iAs concentration considering the different group of rice-based beverages.

As evidenced by the scatter plot (Figure 4), no statistically significant correlation was observed between the declared rice content and iAs concentration (Pearson correlation coefficient = 0.238), even when considering the different groups. Given the limited information of raw materials characteristics (e.g., provenance, seasonality) and processing methods, as well as the limited number or commercially available alternatives for several groups, further considerations based on uni- or multivariate analyses cannot be advanced. However, an in-depth literature search revealed that other vegetable drinks are not (or minimally) a source of exposure to iAs [26,27]. It can be assumed with sufficient confidence that most of As comes from rice. In fact, the two main As species in rice are iAs and DMA with a relative prevalence in grain depending on many different factors, such as plant physiology and genetics, soil characteristics, paddy management practices [29].

**Figure 5:**
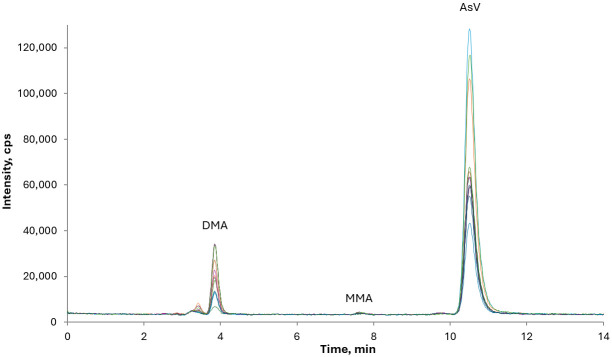
Representative merged chromatograms of rice-based beverage samples showing the distribution of iAs and oAs species (DMA and MMA).

The merged chromatographic profiles of representativesamples are reported in Figure 5. None of the samples exceeded 30 µg kg^−1^ [14]. Although no maximum limits have been established for DMA and MMA, these compounds received particular attention at the recent meeting of the Joint Food and Agriculture Organization of the United Nations (FAO)/World Health Organization (WHO) Expert Committee on Food Additives (JECFA) in October 2025, in relation to new toxicological evidence. Indeed, the Committee recommended the collection of additional occurrence and dietary exposure data [30].

In this reshaping context, the occurrence data generated in this study are of particular significance when compared with other studies on this matrix. Meharg et al. determined DMA, MMA, and iAs in rice milk produced in European countries [31]; Pedron et al. provided speciation results for rice milk samples produced in Italy [32]; dos Santos et al. analyzed rice milk powder from Brazil [33]; Guillod-Magnin et al. determined iAs in rice drink from Switzerland [34]; Munera-Picazo et al. reported results for rice milk from Spain [35]; Signes-Pastor et al. provided information about tAs and arsenic species in a Japanese fermented sweet rice drink, amazake [36].

The results are comparable with other studies carried out worldwide (Table 4). In details, iAs levels observed in the present study are consistent with those reported in Spain and Italy. However, they are slightly lower than the values reported in earlier surveys in Brazil and Switzerland. The latter is particularly relevant since it is centered on toddlers. Furthermore, the present study encompasses the largest number of samples analyzed to date, with the exception of those considered in the most recent EFSA report. Nevertheless, it is worth noting that this report is characterized by a high proportion of left-censored data (42%) for iAs, as well as a limited number of samples (n = 14) in which both iAs and tAs were quantitatively determined [8].

### 3.3. Dietary Exposure and Risk Characterization

Dietary exposure to iAs from rice-based beverages was estimated by combining the occurrence data generated in this study with individual consumption records from the most recent Italian national dietary surveys (IV SCAI CHILD and IV SCAI ADULT). Consumption levels were low in the overall population but showed clear differences between all subjects and consumers only (Table 5). The data for all subjects and consumers are taken into consideration, as well as the consumption data for the European population with the range min-max. These findings are consistent with Italian consumption data, suggesting that the risk characterization for the Italian population may be analogous to that of the European population.

Toddlers were by far the highest consumers among those reporting intake (18.0 g kg^−1^ bw per day), followed by other children (8.51 g kg^−1^ bw per day), whereas adolescents, adults, and the elderly showed markedly lower consumption. Although the number of consumers in the Italian survey is generally low, the average consumption of rice drinks among Italian consumers by the age classes is comparable to that of consumers in the other European surveys. For Italian adolescent consumers, the consumption of rice-based drinks is slightly higher than in other European countries but that does not have a substantial impact on the exposure assessment.

Based on these consumption levels and the measured mean concentration of iAs in rice drinks (15 µg kg^−1^), chronic exposure estimates for the total population ranged from 0.0001 µg kg^−1^ bw per day in the elderly to 0.0042 µg kg^−1^ bw per day in toddlers (Table 6). Among consumers only, the exposure increased considerably, reaching 0.27 µg kg^−1^ bw per day for toddlers and 0.13 µg kg^−1^ bw per day for other children. Although these values remain lower than the overall chronic dietary exposure to iAs estimated by EFSA for Italy (0.03–0.61 µg kg^−1^ bw per day, depending on age group), they represent a non-negligible contribution for regular consumers, particularly for younger age classes disproportionately reliant on plant-based beverages.

Risk characterization was carried out using the MOE approach, applying the RP (0.06 µg kg^−1^ bw per day) selected by EFSA from human epidemiological data. In the total population, MOE values ranged from 14 in toddlers to 598 in the elderly, mirroring their respective consumption levels. Among consumers only, the MOE decreased substantially in the youngest age groups, reaching very low values in toddlers (MOE = 0.2) and other children (MOE = 0.5), raising a potential health concern, despite the uncertainties.

These data should also be interpreted in the broader context of evolving dietary habits. The increasing diversification of food choices, including the rice in consumption of plant-based beverages and products marketed for specific nutritional needs, may significantly influence exposure patterns to arsenic. As the intake of plant-derived commodities continues to expand, the relative contribution of rice drinks to total iAs exposure may become more relevant, particularly for children, vegans/vegetarians and flexitarians, individuals with coeliac disease or gluten intolerance, and other groups relying on milk alternatives [41].

Previous EFSA assessments noted that the impact of rice drinks on overall dietary exposure was likely underestimated due to the high proportion of left-censored data (42%) and the uncertainty associated with substitution methods (LB–UB differences). In addition, for these relatively new consumed foods, an extra uncertainty factor should be considered, i.e., limited consumption information and available surveys, especially for some age groups (e.g., toddlers n = 5; other children n = 2). Despite these uncertainties and gaps, the present dataset contributes to reducing this uncertainty by providing robust, uncensored occurrence data and population-specific exposure estimates.

To sum up, consumer exposure to iAs in food remains a health concern, according to the EFSA latest risk assessment report. The results of this study confirm that rice-based beverages, although contributing modestly to total population exposure, may pose a more meaningful risk for specific subgroups—particularly toddlers and young children with high consumption patterns. In such cases, a reduction in the consumption of rice-based beverages, coupled with a diversification of plant-based beverage options, may serve to mitigate exposure to iAs.

## 4. Conclusions

This study provides an updated overview of iAs and oAs occurring in rice-based beverages available on the Italian market. The analytical method, developed and validated in accordance with UNI EN 16802:2016, proved suitable for the simultaneous determination of iAs, AB, DMA, and MMA, while also demonstrating sufficient sensitivity to generate robust occurrence data without left censoring for iAs. In addition, tAs was determined by a validated ICP-MS method. The contamination levels were found to be homogeneous across products, with iAs representing the predominant species. Furthermore, all samples complied with the current European maximum level for non-alcoholic rice-based drinks.

When combined with recent Italian consumption data, these occurrence data enabled a general population and age-specific assessment of dietary exposure. While rice-based beverages contribute minimally to overall population exposure, intake estimates for habitual consumers, especially during early childhood, indicate narrow margins of exposure, suggesting potential risk. This finding lends further support to the mounting concern for vulnerable subgroups, who may consume rice drinks as substitutes for milk. The increasing diversification of dietary habits, particularly the expanding use of plant-based beverages among individuals with specific nutritional needs, highlights the need for continuous monitoring of iAs in these products. Finally, continued efforts in analytical method optimization, database improvement, consumer guidance, and effective risk communication policies will contribute to more accurate exposure and risk assessment, and, more importantly, better protection of public health.

## Figures and Tables

**Table 1:** Instrumental operational conditions.

ICP-MS Parameters	
ICP RF Power	1600 W
Nebulizer Gas Flow STD/KED	1.03 L min^−1^
Plasma Gas flow (Argon)	15 L min^−1^
Auxiliary Gas flow (Argon)	1.2 L min^−1^
Helium KED Gas Flow	4.20 mL min^−1^
Internal standards	^103^Rh
Analytical masses	^75^As (STD), ^75^As (KED), ^35^Cl
RPq	0.25 (0.8 for ^35^Cl, HPLC)
Dwell time	50 ms (for HPLC 750 ms and 250 ms for As and Cl, respectively)
Flow rate	1.0 mL min^−1^
**Chromatographic conditions**	
Column	Hamilton PRP–X100 (4.6 × 250 mm, 5 µm) (Reno, NV, USA)
Column temperature	18 °C
Injection volume	50 µL
Mobile Phase	35 mMol (NH_4_)_2_CO_3_ in 3% (*v*/*v*) CH_3_OH, pH 10.3 (adjusted with aqueous ammonia)
Flow rate	1.0 mL min^−1^
Elution	Isocratic, 12 min
Arsenic species	AB, DMA, MMA, As(V)

**Table 2:** Trueness assessment. Concentrations expressed as μg kg^−1^ (mean ± SD) for total As and As species.

	Total As	DMA	MMA	iAs	Σ Species	Mass Balance ^1^ (%)
ERM-BC211 *(n = 12)*	262 ± 7 *(260 ± 13) ^2^*	124 ± 3 *(119 ± 13) ^2^*	17 ± 2	123 ± 6 *(124 ± 11) ^2^*	264 ± 8	101
NIST SRM 1568b *(n = 6)*	292 ± 8 *(285 ± 14) ^2^*	179 ± 2 *(180 ± 12) ^2^*	11.0 ± 0.2 (*11.6 ± 3.5*) ^2^	99 ± 1 *(92 ± 10) ^2^*	89 ± 3	99
NMIJ CRM 7532-a *(n = 12)*	321 ± 6 *(320 ± 10) ^2^*	17.8 ± 2.2 *(18.6 ± 0.8) ^2^*	3 ± 1	301 ± 24 *(298 ± 8) ^2^*	322 ± 26	100

^1^ Ratio of the sum of As species in the extract to total As. ^2^ Certified reference value.

**Table 3:** Mean occurrence levels of total arsenic and species (μg kg^−1^) in rice drinks available on Italian market. The percentage of rice reported on the product labels is also indicated.

	% Rice	tAs	Σ Species	Recovery * %	DMA	MMA	iAs	% iAs **
Rice and Almond								
A	14	24	20	83	3	<LOQ	17	85
B	16	58	43	74	28	0.9	14	33
Rice hazelnut and cocoa								
A	14	20	18	90	5	<LOQ	13	72
Rice and coconut								
A	14	20	15	75	2	<LOQ	13	87
B	17	21	22	105	2	<LOQ	20	91
C	17	25	24	96	2	<LOQ	22	92
D ***	7.7	9	8	89	1	<LOQ	7	88
Brown rice								
A	14	20	19	97	2	<LOQ	17	89
B	17	16	14	85	1	<LOQ	13	96
Rice								
A	17	22	21	95	4	<LOQ	17	81
B	17	19	18	95	3	<LOQ	15	83
C ***	17	22	21	95	3	<LOQ	18	86
D	14	20	21	105	3	<LOQ	18	86
E	14	23	22	96	3	<LOQ	19	86
F	17	17	18	106	3	<LOQ	15	83
G	14	18	14	79	3	<LOQ	11	76
H	16	10	9	87	1	<LOQ	8	89
I	17	18	15	84	2	<LOQ	13	90
L	14	14	12	88	2	<LOQ	11	85
M	17	25	23	92	2	0.7	21	90
N	12	21	24	113	1	<LOQ	23	96
O	13.7	33	28	84	3	0.6	24	85
P	14	16	14	91	2	<LOQ	12	83
Q	14	15	13	89	1	0.6	11	87
R ****	17	20	15	77	3	<LOQ	13	82
Mean	15	23	21	91	3	1	15	84
Median	14	22	20	90	2	1	15	86
Min–max	7.7–17	9–58	8–43	74–113	1–28	<LOQ-1	7–24	33–97

* Ratio of the sum of As species to tAs. ** Proportion of iAs as compared to the sum of As species. *** As declared on the label, the sample was enriched with calcium and vitamins. **** The sample contains seaweed (*Lithothamnion*) among the ingredients listed on the label.

**Table 4:** Occurrence levels of iAs, MMA, DMA, and tAs in non-alcoholic rice-based beverages reported in the present study and in previously published investigations.

N	iAs	MMA	DMA	tAs	Ref.
25	7–24	1	1–28	9–58	This study
9	12.8 ± 0.4			29.5 ± 1.1	[26]
4	10.22–19.47	<LOD-22.57	<LOD-1.24	14.57–46.94	[37]
15	7.1–20.7	<LOD-0.82	1.1–12.7	10.2–33.2	[31]
6	4.3–15.6			4.3–20.3	[38]
15	4.8–34.0	0.3–0.8	0.4–9.8	5.2–28.0	[34]
4	7.3–17.2	<LOD	<LOD-6.2	8.4–18.9	[35]
3	16.8–26.6			16.4–57.0	[32]
-	13.1			16.6	[39]
43 *	8–17				[8]

* 42% left censored data (LC).

**Table 5:** Chronic consumption of rice-based beverages (g kg^−1^ bw per day) in the Italian population, reported for all subjects and consumers only, by age class, based on data from the IV SCAI CHILD and IV SCAI ADULT dietary surveys, compared with corresponding European surveys.

			Chronic Rice Drink Consumption (g kg^−1^ bw per day)				
			Italy			Europe	
			Mean			Mean (Min–Max)	
		N *	All Subjects	Consumers	N * (Range Survey)	All Subjects	Consumers
Toddler		5	0.28	18.0	53 (1–22)	0.13 (0.01–0.50)	13.6 (3.36–19.3)
Other children		2	0.05	8.51	64 (1–13)	0.05 (0.0–0.19)	7.67 (0.02–18.2)
Adolescents		2	0.04	4.95	33 (1–8)	0.02 (0.0–0.05)	2.84 (1.15–4.95)
Adults		16	0.05	2.07	119 (1–27)	0.01 (0.0–0.05)	1.91 (0.01–2.90)
Elderly		1	0.01	1.04	20 (1–4)	0.01 (0.0–0.03)	1.37 (0.49–3.31)

* Number of consumers.

**Table 6:** Chronic dietary exposure (lower and upper bound mean estimates) of the Italian population to iAs (µg kg^−1^ bw per day) by age classes (total population) alongside contribution and margin of exposure of rice drinks for total population and consumers only.

	Italian Dietary Exposure (µg kg^−1^ bw per day)					
	Rice Drink *				Total Diet **	
	All Subject		Consumers		All Subject	
	Mean	MOE	Mean	MOE	Mean_LB	Mean_UB
Toddler	0.0042	14	0.27	0.2	0.3	0.61
Other children	0.0008	80	0.13	0.5	0.15	0.32
Adolescents	0.0005	112	0.07	0.8	0.08	0.16
Adults	0.0007	87	0.03	1.9	0.03	0.12
Elderly	0.0001	598	0.02	3.8	0.06	0.11

* Data from Italian national dietary surveys (IV SCAI CHILD and IV SCAI ADULT) in EFSA Comprehensive European Food Consumption Database. ** EFSA latest chronic dietary exposure assessment [8] used INRAN SCAI 2005-06 Italian survey [40].

## Data Availability

The original contributions presented in this study are included in this article. Further inquiries can be directed to the corresponding authors.

## Abbreviations

The following abbreviations are used in this manuscript:

AB	Arsenobetaine
AC	Arsenocholine
As	Arsenic
As(III)	Arsenite
As(V)	Arsenate
BMDL05	Benchmark Dose Lower Confidence Limit for a 5% increase in response
bw	Body weight
CRM	Certified Reference Material
DMA	Dimethylarsinic acid
EC	European Commission
EFSA	European Food Safety Authority
FAO	Food and Agriculture Organization of the United Nations
FoodEx2	Food classification and description system developed by EFSA
HPLC	High-Performance Liquid Chromatography
HPLC-ICP-MS	High-Performance Liquid Chromatography coupled with Inductively Coupled Plasma Mass Spectrometry
IARC	International Agency for Research on Cancer
ICP-MS	Inductively Coupled Plasma Mass Spectrometry
iAs	Inorganic arsenic
JECFA	Joint FAO/WHO Expert Committee on Food Additives
KED	Kinetic Energy Discrimination
LB	Lower Bound
LOD	Limit of Detection
LOQ	Limit of Quantification
MB	Middle Bound
MOE	Margin of Exposure
MMA	Monomethylarsonic acid
oAs	Organic arsenic
RP	Reference Point
RSD	Relative Standard Deviation
tAs	Total arsenic
UB	Upper Bound
WHO	World Health Organization
